# miRNA-mRNA Regulatory Network Reveals miRNAs in HCT116 in Response to Folic Acid Deficiency via Regulating Vital Genes of Endoplasmic Reticulum Stress Pathway

**DOI:** 10.1155/2021/6650181

**Published:** 2021-04-26

**Authors:** Lin Lu, Hongbo Zhao, Jianjun Liu, Yuwen Zhang, Xu Wang

**Affiliations:** ^1^School of Life Sciences, Yunnan University, Kunming 650504, China; ^2^Yunnan Key Laboratory of Stem Cell and Regenerative Medicine, Biomedical Engineering Research Center, Kunming Medical University, Kunming 650500, China; ^3^Science and Technology Achievement Incubation Center, Kunming Medical University, Kunming 650500, China; ^4^School of Life Sciences, The Engineering Research Center of Sustainable Development and Utilization of Biomass Energy, Yunnan Normal University, Kunming 650500, China

## Abstract

Moderate folic acid (FA) intake is an effective strategy that slows colorectal cancer (CRC) progression. However, high consumption of FA may trigger the transition of precancerous tissue towards malignancy. MicroRNAs (miRNAs) are considered to be potential biomarkers of CRC. Thus, identification of miRNAs of dysregulated genes in CRC cells by detailed analysis of mRNA and miRNA expression profile in the context of FA deficiency could substantially increase our understanding of its oncogenesis. mRNA-seq and miRNA-seq analyses were utilized to investigate the expression of miRNAs in FA-deficient CRC cell line–HCT116 through massive parallel sequencing technology. A total of 38 mRNAs and 168 miRNAs were identified to be differentially expressed between CRC groups with or without FA deficiency. We constructed an miRNA-mRNA network for the vital regulatory miRNAs altered in FA-deficient CRC cells. The mRNAs and miRNAs validated by Western blotting and RT-qPCR were consistent with the sequencing results. Results showed that FA deficiency upregulated some miRNAs thereby inhibiting the expression of critical genes in the endoplasmic reticulum (ER) stress pathway. Dysregulated miRNAs in our miRNA-mRNA network could contribute to CRC cell in response to deficient FA. This work reveals novel molecular targets that are likely to provide therapeutic interventions for CRC.

## 1. Introduction

Colorectal cancer (CRC) is one of the most common malignancies worldwide and a complex and heterogeneous disease with multiple risk factors responsible for its high incidence and mortality, including genetic, epigenetic, and lifestyle risks [1–3]. Interestingly, low vegetable diet is thought to be an important contributor factor to the high incidence and mortality of CRC in China [4]; also, leafy green vegetables are sought to the main sources of folate in the diet.

Folic acid (FA) is a water-soluble form of vitamin B9 and occurs in a variety of derivative styles. FA plays a critical role in de novo synthesis of nucleic acid synthesis and biological methylation [5], and it is related to maintaining the health of the human genome. FA deficiency is therefore hypothesized to contribute to CRC through disruption of these processes [6, 7], and moderate amounts of FA can protect the normal colorectal mucosa [8, 9]. Inversely, the excessive intake of FA may accelerate the progression of precancerous tissues to malignancy [10–12]. However, the molecular mechanisms through which FA influences CRC development remain unclear.

In principle, dysregulated oncogenes and tumor suppressors might be considered CRC apparent biomarkers. In addition, microRNAs (miRNAs), a class of small noncording RNA with a length of ~22 nucleotides known to modulate gene expression [13–15], are aberrantly expressed in most human cancers, including CRC [16]. They involve in fundamental cancer progression such as proliferation, apoptosis, invasion, angiogenesis, and other biological processes, which have been examined for their potential role as CRC diagnostic, prognostic, and therapeutic biomarkers [17]. Consequently, miRNAs are valuable oncogenic or tumor suppressing roles in CRC development.

With the advent of next-generation sequencing as an effective method for characterizing the mRNA and miRNA expression at the whole-genome scale, it is now feasible to profile gene or miRNA expression at a particular moment. There is necessary to better define how specific miRNAs can modulate deficient FA response of CRC and possibly interfere with multiple genes and pathways implicated in the phenomenon.

In this study, we used high-throughput sequencing to obtain gene and miRNA expression profiles in the context of deficient vs. adequate concentrations of FA in the CRC cell line (HCT116). We next carried out protein-protein interaction (PPI) analysis and constructed miRNA-mRNA networks to uncover the critical molecules with the potential of serving as diagnostic CRC biomarkers. Our data show that deregulated genes and miRNAs can be used to better understand the roles of FA in CRC and uncover the complex molecular mechanisms by which FA may influence CRC.

## 2. Materials and Methods

### 2.1. Cell Culture

The CRC cell lines HCT116 (ATCC®CCL-247™) and SW620 (ATCC®CCL-227™) used in the present study (tested negative for mycoplasma, bacteria, yeast, and fungi) were obtained from the American type culture collection (ATCC). Cell lines were cultured in two different concentrations of FA: adequate FA levels and FA deficiency state. In FA adequate group, the CRC cell lines were maintained in RPMI 1640 (containing 2260 nmol/L FA, 11875-093, Gibco by Life Technologies™, UK) supplemented with 8% fetal bovine serum, Dialized (30067334, Gibco by Life Technologies™, NZ Origin), 100 mg/L penicillin and 100 mg/L streptomycin (10378016, Gibco by Life Technologies™, NY, USA), and 2 mM GlutaMAX™-1(100X) (35050-061, Gibco by Life Technologies™, France) at 37°C with 5% CO2. In the FA deficient group, a dose of 22.6 nmol/L FA was considered inadequate concentration given that our previous studies showed that 30 nmol/L FA readily induces unstable genome in lymphocytes [18, 19]. In the FA deficient group, the RPMI 1640 containing 2260 nmol/L FA was diluted 100-fold using the RPMI 1640 medium lacking FA (27016-021, Gibco by Life Technologies™, USA). The rest of the ingredients and culture conditions were as in the adequate group.

The CRC cell lines were continuously exposed to deficient/adequate FA media for 4 weeks, and the respective culture media were changed every 3 days. After 28 days of intervention, the CRC cell lines were harvested, washed two times with phosphate-buffered saline (PBS), and digested with 0.25% Trypsin-EDTA. Each group sample was divided into three aliquots that were either used for RNA extraction, sequencing, or RT-qPCR analysis. Meanwhile, HCT116 cells were seeded in 12-well plates (1 × 10^5^/well), and cell proliferations of two groups were observed by cell counts every 3.5 days (5 parallel wells for each group). Until at day 28 of the intervention, proteins were extracted from the remaining cells besides collecting sufficient cells for flow cytometry.

### 2.2. Flow Cytometry for Detecting Cell Apoptosis

Annexin V-FITC Apoptosis Detection Kit (KGA107, KeyGEN BioTECH, China) was used for detection whether inadequate FA could affect the situation of cell apoptosis. At day 28 of intervention, more than 1 × 10^5^ HCT116 cells were digested with EDTA free trypsin (15050065, Gibco by Life Technologies™, USA) and harvested by centrifugation. Then, cells were resuspended in Binding Buffer with Annexin V-FITC and Propidium Iodide (PI). The cell death was detected with a flow cytometer (CyFlow® Space, PARTEC, Germany).

### 2.3. mRNA and miRNA Sequencing

The extraction and sequencing of mRNA and miRNA were performed by BGI Genomics Co., Ltd (Shenzhen, China). Briefly, total RNA was extracted from HCT116 using TRIzol™ reagent (Invitrogen, Carlsbad, USA) for mRNA and miRNA sequencing. The RNA quality and quantity were determined with the RNA Nano 6000 Kit on the Agilent 2100 Bioanalyzer system. The samples were stored at −80°C until further use. In brief, total RNA (10 *μ*g) from each sample was reverse-transcribed to form cDNA libraries for mRNA sequencing. First, 1 *μ*g high-quality total RNA was separated by PAGE gel, and 18-30 nt stripes were selected for recycling. Single-stranded DNA adapter with 5-adenylated and 3-blocked was linked to 3′ of selected small RNAs. Both mRNAs and miRNAs from each sample were sequenced using the BGISEQ-500 platform. Two replicates for each sample were sequenced independently.

### 2.4. Data Analysis

In order to obtain clean reads, raw reads containing adapter sequences and low-quality sequences the using an internal software SOAPnuke and the cleaned-up data stored in FASTQ format. The clean reads of mRNA were then mapped to the UCSC human reference genome (build hg38) using HISAT v2.0.4 (http://www.ccb.jhu.edu/software/hisat). Next, Bowtie2 v2.2.5 (http://bowtie-bio.sourceforge.net/Bowtie2/index.shtml) and RSEM v1.2.12 (http://deweylab.biostat.wisc.edu/RSEM) were used to calculate the expression levels of genes and transcripts. The gene expression was expressed as fragments per kilobase of exon model per million mapped reads (FPKM). We next analyzed differentially expressed genes (DEGs) using the DEGseq algorithms [20]. Genes were classified as differentially expressed relative to reference RNA expression levels if the adjusted *p* values were<0.05 and if the absolute value of log2 ratio was ≥1.

Filtered sequences of miRNAs were aligned using the miRBase (release 22) (http://www.mirbase.org/) and other sRNA databases by AASRA. Small RNA expression levels were calculated using TPM (Transcripts Per Kilobase Million) [21]. For this analysis, *q* values (adjusted *p* values) of <0.01 and an absolute value of |log2 ratio| of >1 were used to establish the significance of differences in the miRNA expression.

### 2.5. GO Annotation and KEGG Pathway Analysis of DEGs

Functional interpretation of the DEGs through Gene Ontology (GO) term and pathway analysis was further done using the clusterProfiler package. For GO analysis, a value threshold of 0.05 (*p*. adjust<0.05) was used as the cutoff threshold for identifying significantly enriched GO terms. In pathway analysis, enrichment analysis was carried out using the hypergeometric test with a cutoff threshold of 0.05 based on the Kyoto encyclopedia of genes and genomes (KEGG) database.

### 2.6. The PPI Network and the miRNA-mRNA Interaction Network

The interactions of proteins expressed by DEGs between deficient/adequate FA in HCT116 were utilized STRING v11.0 (https://string-db.org/) to perform, which were determined by the interaction scores with at least medium confidence. Target miRNAs of DEGs were predicted simultaneously. The target-miRNAs of the DEGs were predicted using miRWalk 2.0 database with 4 different algorithms (RNA22, miRanda, miRWalk, and TargetScan); miRNAs were identified as target-miRNA for respective genes only if they were identified by 3 out of the 4 algorithms [22]. Then, our own predict miRNAs were merged with experimentally confirmed in vivo and in vitro reference data for correlation between target-miRNAs and DEGs from miRTarBase Release 7.0 (http://mirtarbase.mbc.nctu.edu.tw/php/index.php). The interaction networks between DEGs and target-miRNAs were constructed using the Cytoscape (http://cytoscape.org).

### 2.7. RT-qPCR for Detection of DEGs and miRNA Expression Levels

miRNA and mRNA extraction was carried out synchronously using the E.Z.N.A. ™ miRNA Kit (R6842, OMEGA Bio-Tek, USA) according to the manufacturer's protocol. RNA quality and yield were determined using Nano-300 (Allsheng). Reverse transcriptions were then done using the PrimeScript™ RT Reagent Kit with gDNA Eraser (RR047A, Takara, China) and miRcute plus miRNA first-strand cDNA Synthesis Kit (KR211, Tiangen Biotech, Beijing) to synthesize the first-stand cDNA of mRNA and miRNA, respectively, according to the manufacturer's instructions. Primers of mRNAs were designed using the National Center of Biotechnology Information (NCBI)'s Primer Blast (Table 1); forward primers of miRNAs were purchased from Tiangen Biotech. RT-qPCR for the quantified detections of DEGs and miRNAs was done on a BIO-RAD CFX96™ Real-time system using Fast Start Universal SYBR Green Master (Roche, Mannheim, Germany) and miRcute Plus miRNA qPCR detection Kit (SYBR Green) (FP411, Tiangen Biotech, Beijing) severally. U6 and GAPDH served as reference gene controls for miRNA and mRNA detection, respectively. The relative expression levels of DEGs and miRNAs were calculated using the 2^-△△Ct^ method and expressed as mean ± standard errors. Independent sample *t*-test analysis was to estimate statistical significance. Statistical analysis was done using IBM's SPSS statistical suite 22.0. Differences were considered statistically significant if the *p*  was <0.05.

### 2.8. Western Blotting for Verifying DEGs

Protein was extracted from HCT116 cell lysate via the RIPA buffer (89901, Thermo Scientific, USA) with Protease Inhibitor Cocktail (cOmplete, mimi, EDTA-free, Roche, Germany). The supernatant was quantified, and 20 *μ*g of protein was separated via 10% sodium dodecyl sulphate polyacrylamide gel electrophoresis and transferred to polyvinylidene fluoride membranes (03010040001, Roche, USA). Primary antibody dilutions were as follows: HSP90B1/GRP94 (1 : 500, 14700-1-AP), CALR (1 : 500, 10292-1-AP), PDIA3/Erp57 (1 : 2000, 15967-1-AP), PDIA4/Erp72 (1 : 1000, 1472-1-AP), and HERPUD1 (1 : 500, 10813-1-AP) (Proteintech, China); HSPA5/BIP (1 : 1000, 3177, Cell Signaling Technology, USA); and XBP1 (1 : 1000, ab220783) and ATF3 (1 : 1000, ab207432) (Abcam, UK). The relative expression of protein level was quantitated using QuantityOne v4.6.6 (Bio-Rad) and normalized with GAPDH.

## 3. Results

### 3.1. Effects of FA Deficiency on Cell Proliferation Rate and Apoptosis Rate

**Figure 1:**
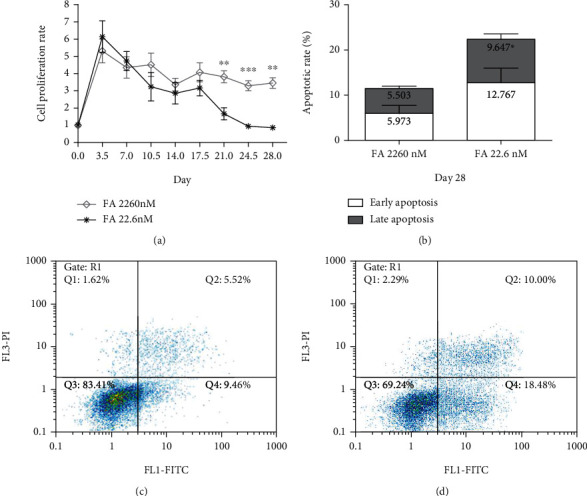
FA deficiency suppressed the proliferation of HCT116 and increased the apoptosis rate at the day 28. (a) Comparison of cell proliferation rate between the group of FA 2260 nmol/L and FA 22.6 nmol/L. (b) Quantitative analysis for the result of apoptosis rate at day 28 of deficient FA intervention via flow cytometry. Flow cytometric analysis using Annexin V-FITC/PI to display one of the apoptosis results in the FA 2260 nmol/L group (c) and the FA 22.6 nmol/L group (d). ^∗∗∗^*p* < 0.001, ^∗∗^*p* < 0.01, ^∗^*p* < 0.05; nM: nmol/L. Data were presented as mean ± SE of three independent experiments.

During the continuous effects of FA deficiency, cell counts demonstrate that 22.6 nmol/L FA could significant restrain the proliferation of HCT116 at day 28 (*p* < 0.01; Figure 1(a)). Flow cytometry results exhibited that the total apoptosis rate of the deficient FA group was increased to 22.414%, including a 12.767% early apoptosis rate and a 9.647% late apoptosis rate, respectively. Both total apoptosis rate and late apoptosis rate in the 22.6 nmol/L FA group were significant higher than the 2260 nmol/L FA group except the early apoptosis rate (*p* < 0.05; Figures 1(b)–1(d)). The early apoptosis rate of the 22.6 nmol/L FA group was higher but had no statistical significance.

### 3.2. Differentially Expressed Genes and miRNAs between Deficient/Adequate Concentrations of FA in HCT116

Next, we analyzed the RNA samples from FA deficient and FA adequate HCT116 cell cultures by high-throughput RNA sequencing. We then applied stringent filters so as to identify the clean mRNA reads and subsequently aligned them to the UCSC human reference genome (hg.38) using hierarchical indexing for spliced alignment of transcripts (HISAT). For miRNA, clean reads were matched to miRBase using alignment-based small RNA annotation (AASRA). Relative to the controls, this analysis identified 38 mRNAs and 168 miRNAs as being differentially expressed FA-deficient HCT116 cells (Table 2). The detailed list of significantly up- and downregulated mRNAs and miRNAs is shown in Table S1 and S2.

### 3.3. GO Enrichment Analysis

**Figure 2:**
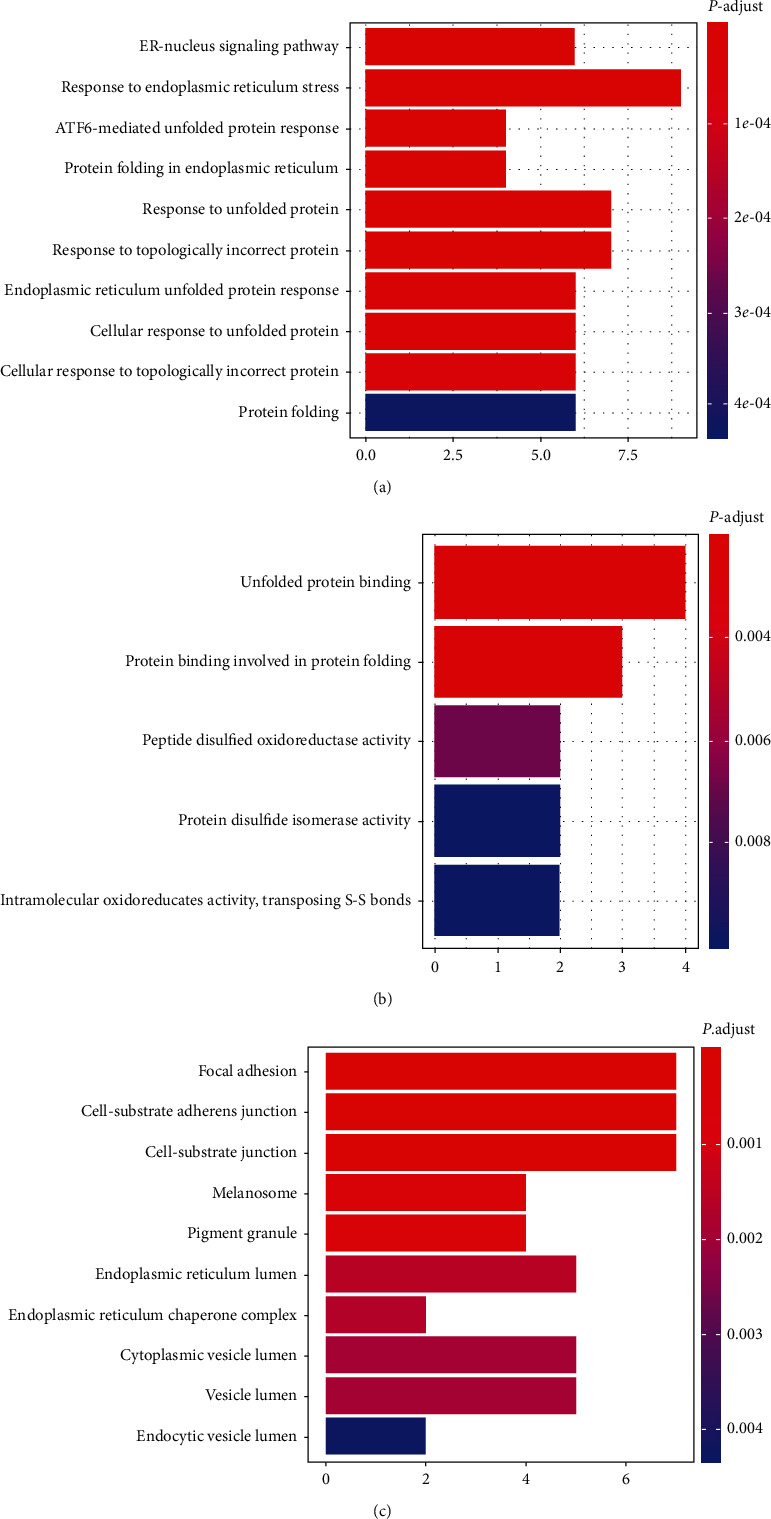
The Gene Ontology term enrichment of DEGs between deficient/adequate concentrations of FA: (a) biological processes of Gene Ontology terms; (b) molecular functions of Gene Ontology terms; (c) cellular components of Gene Ontology terms. The horizontal axis refers to the number of genes in functional analysis, while the vertical axis represents GO term categories. The colors range from blue to red, representing the change in value threshold of identified significantly enriched GO terms (*p*. adjust<0.05) from low to high.

We next performed GO enrichment analysis on the 38 DEGs. From this analysis, 31 GO biological processes (BP) (*p*. adjust<0.05; Table S3) emerged. The top 10 significantly enriched BPs included ER-nucleus signaling pathway, response to endoplasmic reticulum stress, ATF6-mediated unfolded protein response, protein folding in endoplasmic reticulum, response to unfolded protein, response to topologically incorrect protein, endoplasmic reticulum unfolded protein response, cellular response to unfolded protein, cellular response to topologically incorrect protein, and protein folding (Figure 2(a)). Judging from the annotation of BP, FA deficiency in HCT116 may be closely related to endoplasmic reticulum stress (ERS) and unfolded protein response (UPR).

Observation of the enrichment result of the molecular functions (MF) expectedly revealed a strong correlation with UPR (Figure 2(b)). Moreover, focal adhesion, cell-substrate adherens junction, and cell-substrate junction were the highest enrichment in cellular components (CC) (Figure 2(c)).

### 3.4. Pathway Enrichment Analysis

**Figure 3:**
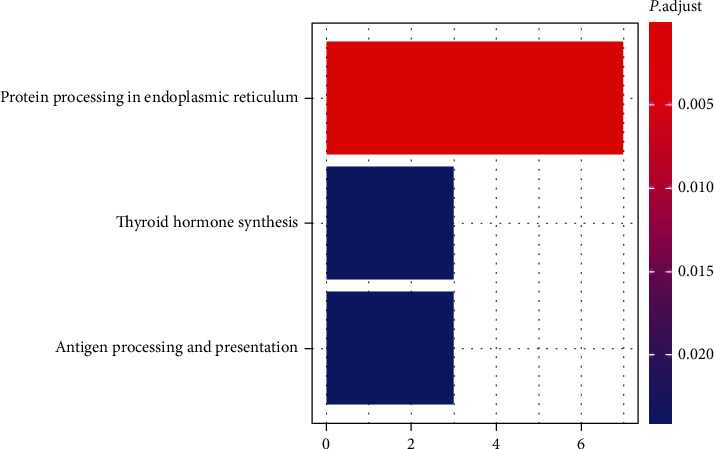
The KEGG analysis of DEGs between deficient/adequate concentrations of FA. The horizontal axis corresponds to the number of annotated differentially expressed genes in pathway analysis, and the vertical axis represents KEGG pathway categories. The colors of each bar represent the value threshold of identified significantly enriched pathways (*p*. adjust<0.05).

We then subjected the DEGs to a pathway enrichment analysis based on the KEGG database and identified protein processing in the endoplasmic reticulum, thyroid hormone synthesis, and antigen processing and presentation as the top 3 pathways that were clearly associated with the downregulated genes (*p*. adjust <0.05; Figure 3). It should be noted *HSPA5* emerged as being in common between the 3 pathways, suggesting that it might be involved multiple pathways in response to FA deficiency in HCT116 (Table 3). In contrast, our analysis could not link any enriched pathway to upregulated genes as there were only 4 upregulated genes in FA deficient/adequate HCT116 cells.

### 3.5. The PPI Network of Protein Processing in DEGs

**Figure 4:**
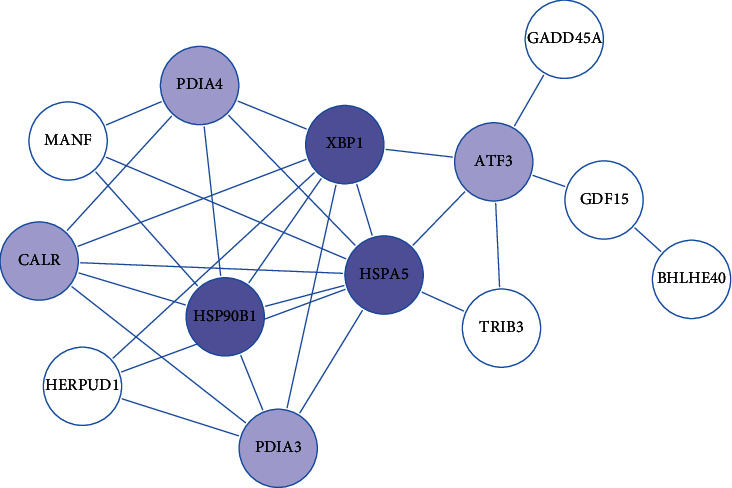
The PPI network of protein processing in DEGs between deficient/adequate concentrations of FA. The blue circles correspond to a critical portion of downregulated DEGs in RNA-seq.

In order to perform their functions, proteins form complexes with other proteins, and often, DEGs within the interaction network frequently have similar biological roles. PPI analysis on the STRING database can elucidate the relationship between known proteins as well as predict interaction interactions between new proteins and known proteins, to excavate the core regulatory genes in the protein network. We therefore analyzed the protein interaction of the 38 DEGs by STRING. This analysis revealed that the more connections there are between proteins the more critical their function together is. This analysis uncovered *HSPA5*, *XBP1*, and *HSP90B1* that seemed to be more crucial than other DEGs in this network. These genes are involved exactly in protein processing pathways (Figure 4). mRNA-seq analysis showed that all genes in this network were downregulation.

### 3.6. Prediction of Target miRNAs of DEGs and Comparison with miRNA-seq

**Figure 5:**
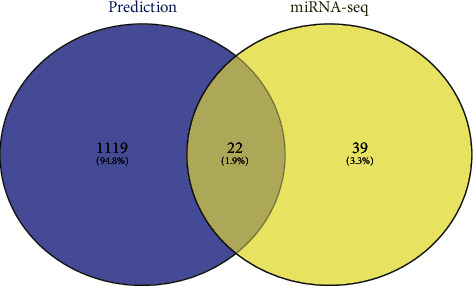
Venn diagram exhibition the intersection between predicted miRNAs by bioinformatics databases and upregulated miRNAs of miRNA-seq. The blue color refers to 1,141 miRNAs that both respond to changes in FA concentration and might target the eight objective genes, while the yellow color shows the 61 upregulated by miRNA-seq.

Based on the results from PPI and KEGG pathway analysis, we selected 7 genes (*CALR*, *HERPUD1*, *HSP90B1*, *HSPA5*, *PDIA3*, *PDIA4*, and *XBP1*) involved in protein processing in the endoplasmic reticulum as well as *ATF3* for prediction of the target-miRNAs of the DEGs through miRWalk2.0 database. First, the miRNA targets of the 8 genes mentioned above were predicted and then combined to obtain a total of 1034 miRNAs. Next, the list of human miRNA target genes was downloaded from miRTarBase, a manually collected and experimentally validated database of miRNA target genes, and merged it with our predicted miRNAs, generating a new set of 1,141 miRNAs. Finally, 22 upregulated miRNAs that were common between the new set of 1,141 miRNAs and the miRNA-seq set of 61 upregulated miRNAs were identified using an online analysis site (http://barc.wi.mit.edu/tools/compare/). Of these, we identified 22 upregulated miRNAs that respond to changes in FA concentration and that might target the selected genes (Figure 5).

### 3.7. Specific miRNA-mRNA Interaction Networks Revealed What Were Pivotal Molecules in FA Deficiency Affecting CRC Cells

**Figure 6:**
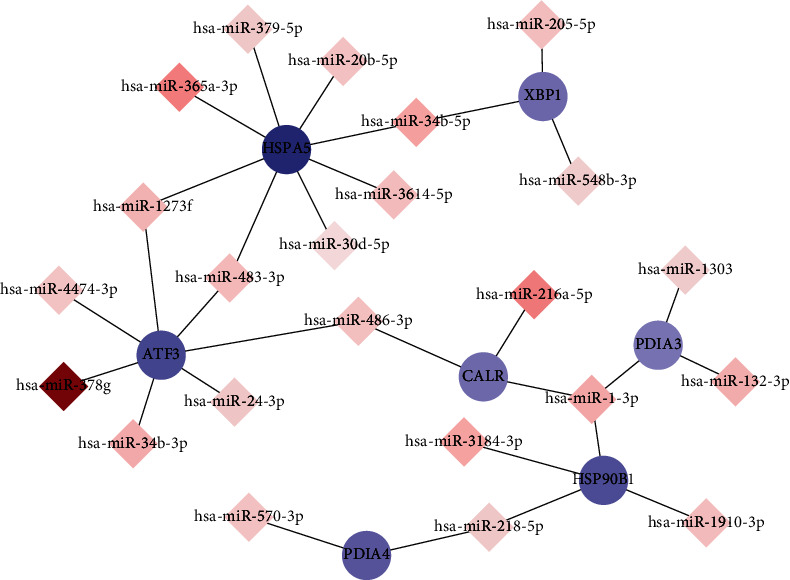
Interaction network between upregulated miRNAs and downregulated mRNAs in FA deficiency affecting CRC cells. Circular nodes represented mRNAs, and diamond nodes expressed miRNAs. Red and blue colors represented upregulation and downregulation, respectively. The darker of the color represents the more significant up/down fold change. Solid lines indicated interaction associations between miRNAs and mRNAs.

To investigate how FA deficiency affects the interaction between miRNA and mRNA in HCT116 cells, the 8 DEGs described above and their target miRNAs were used for the construction of an miRNA-mRNA network. Specifically, 30 miRNA-mRNA pairs with reverse correlation, including 7 downregulated mRNAs and 23 upregulated miRNAs, were visualized using the Cytoscape software (Figure 6). It should be noted that *HERPUD1* was absent from the interaction network (Figure 6), that is because none of the 133 predicted and experimentally validated target miRNAs of *HERPUD1* were consistent with the results of 61 upregulated miRNAs in miRNA-seq. In place of *HERPUD1*, we added mir-30d-5p, which targets *HSPA5*, to the network. miR-30d-5p has been clearly demonstrated to target *HSPA5* [23], and these results are consistent with the predictions of the latest version of TargetScan (TargetScan Human 7.2, http://www.targetscan.org/vert_72/). There was no entry of this miRNA regulating *HSPA5* in the data we downloaded from miRTarBase database. This discrepancy may reflect differences between online analysis tools' algorithms, different database updates, or specific denominated deviation of miRNAs.

Analysis of the miRNA-mRNA network uncovered several critical genes and miRNAs that may affect how FA influences HCT116 cells. For instance, *HSPA5* and *ATF3* occupied the more important sites in the whole governing system because of the more links to miRNAs, while various miRNAs, including miR-1-3p, miR-486-3p, and miR-483-3p, connecting two or more genes, were considered more valuable for research.

### 3.8. Validation of the Expression Level of Vital Genes and miRNAs in FA Deficient/Adequate CRC Cells via RT-qPCR

**Figure 7:**
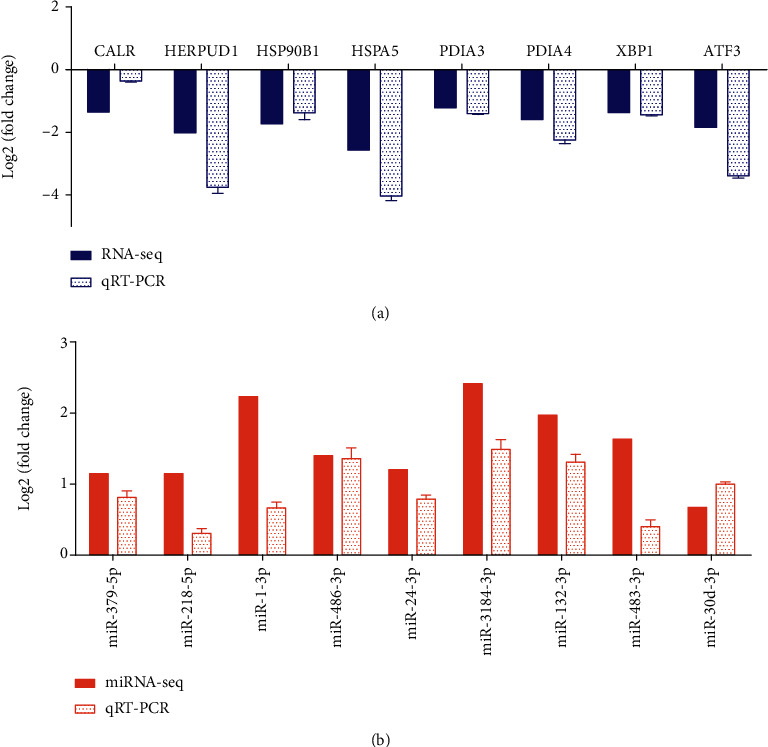
Comparison of the high-throughput sequencing data and RT-qPCR result of HCT116 in FA deficiency: (a) mRNAs; (b) miRNAs. The height of the columns in the chart represents the log-transformed average fold change (deficient FA/adequate FA). All coupled columns between sequencing data and RT-qPCR result have the statistical significance (*p* < 0.05). Bars represent standard errors.

To validate the results of high-throughput sequencing results for genes and miRNAs of interest, we used RT-qPCR to assess their expression in the same samples that we had sequenced. mRNAs (including *CALR*, *HERPUD1*, *HSP90B1*, *HSPA5*, *PDIA3*, *PDIA4*, *XBP1*, and *ATF3*) and miRNAs (including miR-379-5p, miR-218-5p, miR-1-3p, miR-486-3p, miR-24-3p, miR-3184-3p, miR-132-3p, miR-483-3p, miR-378g, and miR-30d-5p) that emerged as differentially expressed following sequencing of FA deficient/adequate HCT116 cells were selected for validation. We observed a strong similarity between the results obtained using the two methods (Figure 7). RT-qPCR results indicated a significant downregulation of the 8 mRNAs, which was completely concordant with the mRNA-seq data (Figure 7(a)). For the miRNAs, with the exceptions of miR-378g, RT-qPCR analysis revealed a significant upregulation, which was almost consistent with the miRNA-seq data, and only statistically significant results of miRNAs can be showed in Figure 7(b). Although the expression of miR-378g was still upregulated, its expression did not significantly (Figure S1(a)).

We then performed similar experiments on a different CRC cell line, SW620, under similar FA conditions and assessed the expression of the same mRNAs and miRNAs assayed in HCT116 cells. RT-qPCR analysis revealed that under FA deficiency, all analyzed genes were significantly downregulated. Likewise, with the exception of miR-378g and miR-218-5p, all miRNAs were significantly upregulated (Figure S2). The expression situation of miR-378g and miR-218-5p in SW620 was consistent with that of miR-378g in HCT116 (Figure S1(b, c)).This analysis recapitulated the observations made in HCT116 cells.

### 3.9. Validation of the Protein Expression of Vital Genes in FA Deficient/Adequate HCT116 Cells

**Figure 8:**
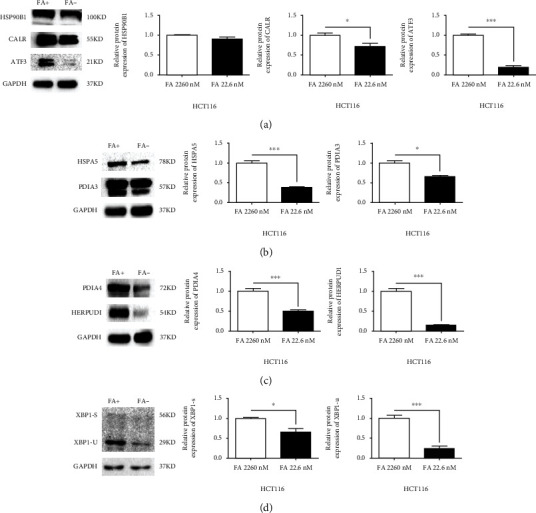
FA deficiency affects the expressions of ER stress associated proteins in HCT116: (a) the expressions of HSP90B1, CALR, and ATF3; (b) the expressions of HSPA5 and PDIA3; (c) the expressions of PDIA4 and HERPUD1; (d) the expressions of XBP1-s and XBP1-u. ^∗∗∗^*p* < 0.001, ^∗^*p* < 0.05. FA+: FA 2260 nmol/L; FA−: FA 22.6 nmol/L; nM: nmol/L. Bars represent standard errors.

The protein expressions of CALR, HERPUD1, HSP90B1, HSPA5, PDIA3, PDIA4, XBP1, and ATF3 were also assessed by Western blotting. It was almost consistent with the RT-qPCR results of HCT116; FA deficiency significantly reduced the expression of these proteins except the HSP90B1 (*p* < 0.05 ~ 0.001, Figure 8), and no significant change in the HSP90B1expression (Figure 8(a)). Both the expression of spliced XBP1 (XBP1-s) and unspliced XBP1 (XBP1-u) were observed in the same time, and XBP1-u was always expressed higher than XBP1-s in FA deficient/adequate HCT116 cells (Figure 8(d)).

## 4. Discussion

Our primary goal of this study was to identify definite miRNAs that regulate abnormally expressed genes in CRC cells in conditions of FA deficiency. We screened a total of 38 mRNAs and 168 miRNAs by high-throughput sequencing, also uncovered novel miRNAs (Table S2) that might not be curated in the current human miRNA catalog or may not have been validated yet. We combined sequencing data analysis with online prediction tools in order to overcome limitations like detection of a limited number of relatively high-abundance transcripts [24]. In the study, the differentially expressed mRNAs were enriched in the pathway of protein processing in the endoplasmic reticulum (ER) by GO enrichment and KEGG analysis, and the 23 miRNAs that negatively regulated mRNAs in aforesaid pathway were identified through constructing the miRNA-mRNA network. Thus, the network has been simplified, and the most critical miRNAs could be easily found.

Different from the previously reported that differentially expressed mRNAs obtained from long-term defective intervention of HCT116 with 0-0.6 nmol/L FA detected by specialized array [25], the pathways related to apoptosis and cancer had not been enriched in our sequencing results. Compared with the works by Novakovic et al., the most of DEGs about apoptosis and cancer in HCT116 had no statistically significant change in RNA sequencing (Table S4). That may due to the varying measures for detecting DEGs and the different inadequate FA concentration. Nevertheless, a long time intervention of 22.6 nmol/L FA could influence the proliferation and enhance the apoptosis rate in HCT116.

Numerous studies have associated ER stress and the UPR with CRC [26]. The ER is extremely sensitive to changes in the intracellular environment and is highly vulnerable to altered Ca^2+^ homeostasis, which causes an accumulation of misfolded or unfolded proteins and triggers the UPR, more generally referred to as ER stress [27]. Genes acting along the ER stress pathway might be good therapeutic candidates for treatment of CRC by targeting ER stress-dependent autophagy and apoptosis [28, 29]. Our result should be noted that inadequate amounts of FA may determine the fate of CRC cells by reducing the expression of genes in ER stress pathway.

The expression of *HSPA5*, *XBP1*, and *HSP90B1* involved in ER stress in PPI network was significantly downregulated in FA deficient HCT116 cells. Reduced *HSPA5* and *XBP1-S* indicates that HCT116 cells may not be in ER stress status or can hardly check their high expression in fewer survived cells after long-term stress. As a key upstream activator of the UPR, heat shock protein family A (Hsp70) member 5 (HSPA5/BIP/GRP78) is broadly regarded as a biomarker for ER stress [30, 31]. Data from GEPIA (Gene Expression Profiling Interactive Analysis, a public database newly developed by the Chinese for cancer and normal gene expression profiling) reveal that HSPA5 is markedly upregulated in CRC. The X-box-protein1 (XBP1) can form its splicing variant XBP1-s via the role of endoribonuclease by IRE1 to splice the XBP1-u upon exposure to ER stress. The expression of XBP1-s is highly in various tumors although its role in CRC is still largely controversial. For example, activity of XBP1-s leads to promote CRC cell proliferation by inhibiting *TAp73* transcriptional activity [32]; but Xbp1 reduces colorectal cancer cell proliferation and stemness by activating PERK signaling in CRC cell LS174T [33]. Studies reported that Heat Shock Protein 90 Beta Family Member 1 (HSP90B1), upregulated in many cancers [34, 35], is a major contributor to evaluate the progression of carcinomas and may be a potential target for colorectal cancer therapy [36]. It is noteworthy that FA deficiency affected HSP90B1 only at the transcriptional level from our results of RT-qPCR and Western blotting. Reducing the high expression of these genes in CRC seems to be advantage to suppress CRC.

The role of miRNAs in CRC might provide some answers to illustrate the mechanism by which FA modulates ER stress genes. MiR-1-3p, an miRNA that is frequently downregulated in CRC, has been reported to suppress CRC via multiple biological processes [37]. MiR-218-5p is thought to be tumor suppressive and capable of negatively regulating *DPH1* in CRC cells [38]. MiR-132-3p was demonstrated to be downregulated in CRC tissues and cells [39], which has been reported to be a molecular marker to predict CRC resistance to 5-Fluorouracil-based chemotherapy [40] and usefully constrains cell growth and metastasis in CRC cells [41]. MiR-379-5p is generally considered to be a tumor suppressor to directly suppress the EMT, proliferation, migration, and invasion of carcinoma cells in multiple tumor [42, 43]; however, there is little research on its role in CRC.

Moreover, the same one aberrant miRNA may have discrepant diagnostic and prognostic value as cancer biomarker. MiR-24-3p, which has oncogenic and tumor suppressor functions in various cancers, has been shown to play a critical role in CRC; however, the role of this miRNA is controversial as it has been shown to inhibit CRC cell proliferation, migration, and invasion *in vitro* [44, 45] but is also associated with poor disease-free and overall survival (OS) of colorectal adenocarcinoma patients [46]. Also, although miR-486-3p is a potential strategy to inhibit cervical cancer progression [47], *KRAS* mutation in CRC was associated with upregulation of miR-486-3p and downregulation of miR-378 [48]. It has been reported that miR-483-3p is overexpressed in large subsets of CRC [49], and tissue miR-483-3p could be used as markers for the detection of CRC [50]. Nevertheless, studies have been reported that the miR-483-3p expression was increased in cancer cell lines from different tissues treated with chemicals of oncosuppressor activity or radiation [49, 51]. The expression of miR-3184 was decreased in hepatocellular carcinoma cell [52], whereas it upregulated in acquired radioresistant colorectal cancer cell lines [53]. High exosomal miR-30d-5p level was associated with metastatic progression in advanced rectal cancer [54], but miR-30d-5p was also suppressed by PVT1which promote metastasis and proliferation in CRC [55].

Recent studies have shown that miRNAs, including miR-21-5p, miR-31-5p, miR-155-5p, let-7a, and let-7c, are associated with CRC [56, 57]. However, our miRNA sequencing results failed to detect these miRNAs, which were either not significantly expressed (e.g., miR-155-5p and let-7c), or their up/downregulation level was very modest (e.g., miR-21-5p and miR-31-5p), because the CRC cell lines were cultivated in an environment with severe FA defects. Compared to these similar researches, our mRNA-miRNA network uncovered multiple upregulated miRNAs that might influence the effect of FA in CRC, suggesting that FA deficiency might upregulate miRNAs that regulate ER stress, inhibiting CRC.

Previous studies have shown that FA deficiency causes gene instability and induces cancerization in normal cells; on the contrary, depletion the FA could inhibit carcinogenesis process in cancer cells. In our works, FA deficiency upregulated some miRNAs thereby restricting the expression of some critical genes in ER stress pathway, and these screened miRNAs almost had a positive effect on CRC inhibition. Our results are consistent with previous reports on the inhibitory effect of FA deficiency in cancer cells.

Although this study found that some miRNAs negatively regulate specific ER stress pathway genes and may provide novel candidates for future studies of the role of miRNA in CRC development and progression, a limitation of this study is the use of one CRC cell line for sequencing. To address this limitation, we used a different CRC cell line (SW620) subjected to the same conditions as HCT116, to validate the sequencing results through RT-qPCR. Similar observations were made using the two cell lines. Further studies are needed to further validate our observations.

## 5. Conclusions

In conclusion, to the best of our knowledge, this is one of the few studies to simultaneously profile the expression of both miRNAs and mRNAs on a genome-wide scale in CRC cells in FA deficient conditions. Here, we identified multiple mRNAs and miRNAs that were differentially expressed in CRC cells in conditions of FA deficiency/adequacy. Functional and pathway enrichment analysis revealed that in our study context, the most significantly affected pathway was ER stress. We constructed an miRNA-mRNA regulatory network and uncovered miRNAs that regulating vital target genes in the ER stress pathway. We hypothesize that FA deficiency might upregulate miR-379-5p, miR-218-5p, miR-1-3p, miR-486-3p, miR-24-3p, miR-3184-3p, miR-132-3p, miR-483-3p, and miR-30d-5p with their unique function to inhibit the expression of key genes in the ER stress pathway.

## Figures and Tables

**Table 1:** Primer sequences for the quantitative real-time polymerase chain reaction.

Gene names	NCBI ID	Primer sequences(5′-3′)
*CALR*	NM_004343.3	S: CGCTTTTATGCTCTGTCGGC A: CCACAGATGTCGGGACCAAA
*HERPUD1*	NM_001010989.2	S: GCATCAGGGGCTTTTGTTCC A: TTCCACAATAGGGCCACCTT
*HSP90B1*	NM_003299.2	S: GCCAGTTTGGTGTCGGTTTC A: GGGTAATTGTCGTTCCCCGT
*HSPA5*	NM_005347.4	S: GGAACCATCCCGTGGCATAA A: TGGTAGGCACCACTGTGTTC
*PDIA3*	NM_005313.4	S: AGAGAGCAATGATGGGCCTG A: GTCTTTGCTGAGCTTCTCGC
*PDIA4*	NM_004911.4	S: GCGAGTTTGTCACTGCTTTC A: CGGGTCCCTTGTTGTTCTT
*XBP1*	NM_005080.3	S: AGATCGAAAGAAGGCTCGAATG A: GCTGTCTTAACTCCTGGTTCTC
*ATF3*	NM_001030287.3	S: CTGGAAAGTGTGAATGCTGAAC A: ATTCTGAGCCCGGACAATAC

**Table 2:** Differentially expressed numbers of mRNAs and miRNAs between deficient/adequate concentrations of FA in HCT116 by high-throughput sequencing.

Type	mRNA	miRNA
Up	4	61
Down	34	107
Total	38	168

**Table 3:** Pathway enrichment of DEGs between deficient/adequate concentrations of FA.

ID	Description	*p* value	*p*. adjust	Gene ID
hsa04141	Protein processing in endoplasmic reticulum	1.24E-05	1.11E-05	CALR/HERPUD1/HSP90B1/HSPA5/PDIA3/PDIA4/XBP1
hsa04918	Thyroid hormone synthesis	0.024927715	0.022368269	HSP90B1/HSPA5/PDIA4
hsa04612	Antigen processing and presentation	0.024927715	0.022368269	CALR/HSPA5/PDIA3

*p* value: statistical significance; *p*. adjust: adjusted *p* value.

## Data Availability

The mRNA-seq and miRNA-seq data from this study have been deposited in the NCBI sequence read archive under the BioProject number PRJNA609604, with the fq files spanning accession numbers SRR11240236-SRR11240243 (https://dataview.ncbi.nlm.nih.gov/object/PRJNA609604?reviewer=60p8dbdruor7pr3elbqu64iikh). The data supporting the research results can be obtained from the corresponding authors according to reasonable requirements.

## Supplementary Materials

Supplementary Materials Table S1: differentially expressed mRNAs between deficient/adequate concentrations of FA. Table S2: differentially expressed miRNAs between deficient/adequate concentrations of FA. Table S3: the integrated list about biological processes (BP) of Gene Ontology terms from DEGs between deficient/adequate concentrations of FA. Table S4: summary the expression of several genes affected by FA deficiency in apoptotic pathway and cancer pathway. Figure S1: the supplemental RT-qPCR result of miRNAs in HCT116 and SW620 in FA deficiency conditions. (a) miR-378g in HCT116; miR-378g (b), and miR218-5p (c) in SW620. Bars represent standard errors. Figure S2: the RT-qPCR result of SW620 under the same FA deficiency conditions. (a) mRNAs. (b) miRNAs. The height of the columns in the chart represents the log-transformed average fold change (deficient FA/adequate FA). All columns have statistical significance (*p* < 0.05) in this diagram. Bars represent standard errors.
