# Supplementary material for: miRNA-mRNA Regulatory Network Reveals miRNAs in HCT116 in Response to Folic Acid Deficiency via Regulating Vital Genes of Endoplasmic Reticulum Stress Pathway
Source: Biomed Res Int. 2021 Apr 26;2021:6650181. doi: 10.1155/2021/6650181 (PMC8096553; doi:10.1155/2021/6650181)
Supplement: Supplementary Materials — Table S1: differentially expressed mRNAs between deficient/adequate concentrations of FA. Table S2: differentially expressed miRNAs between deficient/adequate concentrations of FA. Table S3: the integrated list about biological processes (BP) of Gene Ontology terms from DEGs between deficient/adequate concentrations of FA. Table S4: summary the expression of several genes affected by FA deficiency in apoptotic pathway and cancer pathway. Figure S1: the supplemental RT-qPCR result of miRNAs in HCT116 and SW620 in FA deficiency conditions. (a) miR-378g in HCT116; miR-378g (b), and miR218-5p (c) in SW620. Bars represent standard errors. Figure S2: the RT-qPCR result of SW620 under the same FA deficiency conditions. (a) mRNAs. (b) miRNAs. The height of the columns in the chart represents the log-transformed average fold change (deficient FA/adequate FA). All columns have statistical significance (p < 0.05) in this diagram. Bars represent standard errors. [file 6650181.f1.doc]

## Supplementary Materials

Table S1:Differentially expressed mRNAs between deficient/adequate concentrations of FA.

| Gene ID | Symbol | log2 Fold Change | P value |
| --- | --- | --- | --- |
| Up-regulated mRNA | | | |
| 100132287 | LOC100132287 | 3.52644 | 2.61E-11 |
| 101954271 | RNU6-9 | 2.631751 | 1.33E-06 |
| 103625684 | RNU6-2 | 2.631751 | 1.33E-06 |
| 100528016 | TMX2-CTNND1 | 2.089321 | 1.47E-05 |
| Down-regulated mRNA | | | |
| 2923 | PDIA3 | -1.21765 | 8.19E-06 |
| 50854 | C6orf48 | -1.22643 | 1.40E-05 |
| 10263 | CDK2AP2 | -1.25654 | 1.88E-05 |
| 5202 | PFDN2 | -1.32142 | 2.85E-05 |
| 9181 | ARHGEF2 | -1.32638 | 4.06E-05 |
| 811 | CALR | -1.35267 | 2.02E-06 |
| 1647 | GADD45A | -1.35599 | 5.55E-05 |
| 7494 | XBP1 | -1.36996 | 5.91E-06 |
| 3855 | KRT7 | -1.40297 | 1.47E-05 |
| 6385 | SDC4 | -1.44367 | 2.23E-05 |
| 9518 | GDF15 | -1.50583 | 4.39E-08 |
| 2512 | FTL | -1.5536 | 2.25E-05 |
| 2274 | FHL2 | -1.57258 | 8.86E-06 |
| 9601 | PDIA4 | -1.59801 | 2.47E-08 |
| 8553 | BHLHE40 | -1.64485 | 5.81E-05 |
| 7873 | MANF | -1.67186 | 6.79E-08 |
| 138639 | PTPDC1 | -1.68056 | 1.26E-06 |
| 81872 | KRTAP2-1 | -1.688 | 7.38E-05 |
| 57761 | TRIB3 | -1.7017 | 3.10E-05 |
| 7184 | HSP90B1 | -1.73198 | 8.48E-09 |
| 730755 | KRTAP2-3 | -1.78536 | 1.76E-05 |
| 5054 | SERPINE1 | -1.82024 | 3.15E-06 |
| 286343 | LURAP1L | -1.82374 | 1.72E-06 |
| 467 | ATF3 | -1.84038 | 8.08E-06 |
| 256979 | SUN3 | -1.91849 | 4.82E-05 |
| 9709 | HERPUD1 | -2.0205 | 1.52E-09 |
| 100526760 | ABHD14A-ACY1 | -2.21119 | 4.05E-05 |
| 3934 | LCN2 | -2.27022 | 1.03E-10 |
| 7378 | UPP1 | -2.30859 | 6.37E-08 |
| 26827 | RNU6-1 | -2.51024 | 4.10E-06 |
| 80763 | SPX | -2.53932 | 5.69E-09 |
| 3309 | HSPA5 | -2.56725 | 2.69E-15 |
| 63915 | BLOC1S5 | -2.78862 | 2.89E-07 |
| 100528007 | BORCS7-ASMT | -3.50537 | 3.80E-11 |

Table S2:Differentially expressed miRNAs between deficient/adequate concentrations of FA.

| miRNA ID | log2Ratio | Q value |
| --- | --- | --- |
| Up-regulated miRNA | | |
| hsa-miR-378g | 8.698214 | 0 |
| hsa-miR-378d | 6.288149 | 0 |
| novel_mir58 | 5.909503 | 6.21E-09 |
| novel_mir162 | 5.440018 | 9.42E-07 |
| novel_mir213 | 5.26314 | 4.46E-06 |
| novel_mir138 | 5.061506 | 2.14E-05 |
| novel_mir267 | 4.739578 | 0.000178 |
| novel_mir46 | 4.646469 | 0.000297 |
| novel_mir30 | 4.646469 | 0.000297 |
| novel_mir237 | 4.646469 | 0.000297 |
| novel_mir61 | 4.546933 | 0.000497 |
| novel_mir301 | 4.546933 | 0.000497 |
| novel_mir306 | 4.546933 | 0.000497 |
| hsa-miR-216a-5p | 3.868861 | 9.94E-08 |
| hsa-miR-365a-3p | 3.80069 | 2.18E-41 |
| hsa-miR-216a-5p | 3.868861 | 9.94E-08 |
| hsa-miR-365a-3p | 3.80069 | 2.18E-41 |
| hsa-miR-509-3p | 3.402543 | 1.21E-07 |
| hsa-miR-103a-3p | 2.945475 | 0 |
| hsa-miR-34b-5p | 2.440018 | 0.000245 |
| hsa-miR-3184-3p | 2.412564 | 0 |
| hsa-miR-1-3p | 2.231431 | 4.20E-06 |
| hsa-miR-548ae-5p | 2.180151 | 9.41E-38 |
| hsa-miR-34b-3p | 2.11809 | 0.000764 |
| hsa-miR-132-3p | 1.97232 | 8.77E-168 |
| hsa-miR-550a-3-5p | 1.970904 | 1.78E-21 |
| hsa-miR-449b-3p | 1.96197 | 0.000888 |
| hsa-miR-1273f | 1.798472 | 2.67E-05 |
| novel_mir71 | 1.769325 | 3.92E-05 |
| hsa-miR-548am-5p | 1.701104 | 7.68E-07 |
| hsa-miR-151a-3p | 1.679327 | 0 |
| hsa-miR-483-3p | 1.631722 | 4.89E-17 |
| hsa-miR-369-5p | 1.569653 | 3.94E-12 |
| novel_mir266 | 1.486812 | 0.00038969 |
| hsa-miR-1910-3p | 1.485821 | 9.83E-08 |
| hsa-miR-3614-5p | 1.465403 | 0.00079614 |
| hsa-miR-142-3p | 1.444122 | 7.89E-12 |
| hsa-miR-363-3p | 1.433065 | 1.42E-13 |
| hsa-miR-5187-5p | 1.41765 | 0.00054315 |
| hsa-miR-205-5p | 1.406224 | 3.40E-20 |
| hsa-miR-486-3p | 1.400776 | 6.61E-17 |
| hsa-miR-409-3p | 1.324541 | 4.33E-06 |
| hsa-miR-570-3p | 1.314272 | 1.63E-08 |
| hsa-miR-20b-5p | 1.285546 | 7.39E-05 |
| novel_mir134 | 1.25054 | 0.00062634 |
| hsa-miR-211-5p | 1.237605 | 7.33E-09 |
| hsa-miR-4474-3p | 1.225005 | 0.0008528 |
| hsa-miR-4423-5p | 1.222079 | 1.27E-19 |
| novel_mir93 | 1.212514 | 1.29E-08 |
| hsa-miR-24-3p | 1.200851 | 0 |
| hsa-miR-4521 | 1.196464 | 6.06E-91 |
| hsa-miR-23b-5p | 1.19448 | 2.36E-08 |
| hsa-miR-184 | 1.182096 | 9.92E-25 |
| hsa-miR-204-5p | 1.174964 | 1.19E-11 |
| hsa-miR-154-5p | 1.170212 | 2.68E-09 |
| hsa-miR-379-5p | 1.148384 | 5.63E-11 |
| hsa-miR-218-5p | 1.145493 | 0 |
| hsa-miR-483-5p | 1.139442 | 2.78E-26 |
| hsa-miR-378f | 1.138008 | 0 |
| novel_mir6 | 1.120286 | 6.81E-12 |
| hsa-miR-885-5p | 1.087501 | 0.00020367 |
| hsa-miR-548b-3p | 1.002612 | 0.00020358 |
| hsa-miR-1303 | 1.000342 | 4.32E-14 |
| Down-regulated miRNA | | |
| hsa-miR-27a-3p | -1.02766 | 0 |
| hsa-miR-671-3p | -1.03803 | 2.48E-06 |
| hsa-miR-3187-3p | -1.04245 | 2.04E-86 |
| hsa-miR-3158-5p | -1.04469 | 3.30E-05 |
| hsa-miR-4664-5p | -1.04743 | 5.19E-05 |
| hsa-miR-29b-3p | -1.05179 | 0 |
| hsa-miR-374a-3p | -1.06778 | 4.75E-22 |
| novel_mir22 | -1.11746 | 8.04E-06 |
| hsa-miR-339-5p | -1.14081 | 2.02E-195 |
| novel_mir231 | -1.15792 | 1.55E-44 |
| hsa-miR-7112-3p | -1.16731 | 5.46E-09 |
| hsa-let-7g-3p | -1.24657 | 3.60E-19 |
| hsa-miR-590-3p | -1.2657 | 0 |
| hsa-miR-101-3p | -1.268 | 1.36E-46 |
| novel_mir247 | -1.27091 | 1.22E-17 |
| hsa-miR-7975 | -1.2722 | 1.62E-08 |
| hsa-miR-15b-3p | -1.28977 | 1.46E-37 |
| novel_mir215 | -1.30375 | 1.19E-32 |
| novel_mir250 | -1.31251 | 5.20E-10 |
| hsa-miR-1277-3p | -1.32455 | 5.28E-07 |
| novel_mir15 | -1.32455 | 1.55E-05 |
| hsa-miR-652-5p | -1.35664 | 3.05E-05 |
| hsa-miR-6850-5p | -1.38753 | 8.03E-07 |
| hsa-miR-4634 | -1.42392 | 3.93E-05 |
| hsa-miR-212-5p | -1.46206 | 0.00015126 |
| hsa-let-7i-3p | -1.48608 | 1.20E-34 |
| novel_mir42 | -1.49489 | 0.00032967 |
| hsa-miR-27b-5p | -1.49696 | 7.28E-17 |
| hsa-miR-15b-5p | -1.49783 | 0 |
| hsa-miR-2277-5p | -1.50143 | 3.77E-05 |
| hsa-miR-103b | -1.50748 | 0 |
| hsa-miR-4783-3p | -1.52346 | 0.00054315 |
| hsa-miR-3158-3p | -1.5473 | 4.67E-06 |
| hsa-miR-3929 | -1.58235 | 1.81E-09 |
| novel_mir257 | -1.60105 | 1.62E-40 |
| hsa-miR-188-5p | -1.63893 | 8.40E-15 |
| hsa-miR-548o-5p | -1.65818 | 6.41E-16 |
| hsa-miR-3176 | -1.68669 | 4.54E-05 |
| hsa-miR-548ap-3p | -1.77343 | 1.08E-52 |
| hsa-miR-15a-3p | -1.775 | 5.54E-09 |
| hsa-miR-33a-5p | -1.81489 | 0 |
| hsa-miR-616-5p | -1.87186 | 6.24E-07 |
| hsa-miR-4492 | -1.98805 | 2.97E-36 |
| novel_mir268 | -1.98912 | 2.14E-14 |
| novel_mir12 | -1.99739 | 2.47E-10 |
| hsa-miR-4730 | -2.02596 | 4.00E-05 |
| novel_mir311 | -2.05793 | 7.28E-10 |
| novel_mir156 | -2.06653 | 3.05E-70 |
| hsa-miR-7977 | -2.07349 | 1.30E-31 |
| hsa-let-7f-2-3p | -2.28199 | 7.83E-47 |
| novel_mir43 | -2.29605 | 9.42E-07 |
| hsa-miR-6813-3p | -2.33842 | 1.85E-06 |
| novel_mir105 | -2.3559 | 2.73E-58 |
| hsa-miR-365b-3p | -2.42089 | 2.57E-34 |
| hsa-miR-4485-3p | -2.66641 | 9.45E-10 |
| hsa-miR-7108-5p | -2.81842 | 2.90E-10 |
| hsa-miR-4284 | -2.91017 | 2.31E-142 |
| hsa-miR-6723-5p | -2.92339 | 1.04E-14 |
| hsa-miR-423-5p | -2.95364 | 0 |
| novel_mir1 | -3.04178 | 1.59E-21 |
| novel_mir233 | -3.26042 | 1.65E-18 |
| hsa-miR-1307-5p | -3.61314 | 1.04E-112 |
| hsa-miR-4717-3p | -3.84538 | 0.00075838 |
| hsa-miR-4485-5p | -4.1184 | 6.20E-08 |
| hsa-miR-7704 | -4.17228 | 1.97E-206 |
| hsa-miR-378i | -4.25181 | 7.70E-160 |
| hsa-miR-378b | -4.44555 | 0 |
| hsa-miR-550b-3p | -4.50835 | 2.49E-38 |
| novel_mir52 | -4.59171 | 0 |
| novel_mir248 | -4.71985 | 0.00066639 |
| novel_mir16 | -4.71985 | 0.00066639 |
| novel_mir223 | -4.71985 | 0.00066639 |
| novel_mir153 | -4.71985 | 0.00066639 |
| novel_mir168 | -4.84538 | 0.00035517 |
| novel_mir159 | -4.84538 | 0.00035517 |
| novel_mir60 | -4.84538 | 0.00035517 |
| novel_mir23 | -4.84538 | 0.00035517 |
| novel_mir135 | -4.84538 | 0.00035517 |
| novel_mir176 | -4.84538 | 0.00035517 |
| novel_mir216 | -4.84538 | 0.00035517 |
| novel_mir163 | -4.84538 | 0.00035517 |
| novel_mir220 | -4.84538 | 0.00035517 |
| novel_mir308 | -4.96086 | 0.00019269 |
| novel_mir279 | -4.96086 | 0.00019269 |
| novel_mir226 | -4.96086 | 0.00019269 |
| novel_mir143 | -4.96086 | 0.00019269 |
| novel_mir296 | -5.26042 | 2.98E-05 |
| novel_mir284 | -5.26042 | 2.98E-05 |
| novel_mir214 | -5.34788 | 1.60E-05 |
| novel_mir170 | -5.34788 | 1.60E-05 |
| novel_mir139 | -5.34788 | 1.60E-05 |
| novel_mir199 | -5.50835 | 4.67E-06 |
| novel_mir34 | -5.50835 | 4.67E-06 |
| novel_mir20 | -5.65274 | 1.38E-06 |
| novel_mir189 | -5.65274 | 1.38E-06 |
| novel_mir277 | -5.71985 | 7.68E-07 |
| novel_mir136 | -5.78398 | 4.22E-07 |
| novel_mir180 | -5.84538 | 2.30E-07 |
| novel_mir120 | -6.06778 | 2.16E-08 |
| novel_mir209 | -6.06778 | 2.16E-08 |
| novel_mir234 | -6.16731 | 6.84E-09 |
| novel_mir131 | -6.34788 | 7.06E-10 |
| novel_mir291 | -6.58235 | 2.45E-11 |
| novel_mir256 | -6.88905 | 0 |
| novel_mir19 | -7.28279 | 5.04E-17 |
| hsa-miR-378e | -8.55163 | 0 |
| novel_mir141 | -9.43535 | 2.95E-57 |

Table S3:The integrated list about biological processes(BP) of Gene Ontology terms from DEGs between deficient/adequate concentrations of FA.

| ID | Description | p.adjust | Gene ID |
| --- | --- | --- | --- |
| GO:0006984 | ER-nucleus signaling pathway | 1.01E-07 | ATF3/CALR/HERPUD1/HSP90B1/HSPA5/XBP1 |
| GO:0034976 | response to endoplasmic reticulum stress | 2.62E-07 | ATF3/CALR/HERPUD1/HSP90B1/HSPA5/PDIA3/PDIA4/TRIB3/XBP1 |
| GO:0036500 | ATF6-mediated unfolded protein response | 5.00E-07 | CALR/HSP90B1/HSPA5/XBP1 |
| GO:0034975 | protein folding in endoplasmic reticulum | 2.42E-06 | CALR/HSP90B1/HSPA5/PDIA3 |
| GO:0006986 | response to unfolded protein | 3.48E-06 | ATF3/CALR/HERPUD1/HSP90B1/HSPA5/MANF/XBP1 |
| GO:0035966 | response to topologically incorrect protein | 5.82E-06 | ATF3/CALR/HERPUD1/HSP90B1/HSPA5/MANF/XBP1 |
| GO:0030968 | endoplasmic reticulum unfolded protein response | 1.11E-05 | ATF3/CALR/HERPUD1/HSP90B1/HSPA5/XBP1 |
| GO:0034620 | cellular response to unfolded protein | 2.18E-05 | ATF3/CALR/HERPUD1/HSP90B1/HSPA5/XBP1 |
| GO:0035967 | cellular response to topologically incorrect protein | 3.67E-05 | ATF3/CALR/HERPUD1/HSP90B1/HSPA5/XBP1 |
| GO:0006457 | protein folding | 0.000422 | CALR/HSP90B1/HSPA5/PDIA3/PDIA4/PFDN2 |
| GO:0036499 | PERK-mediated unfolded protein response | 0.000557 | ATF3/HERPUD1/HSPA5 |
| GO:0071496 | cellular response to external stimulus | 0.000974 | ATF3/GADD45A/HSP90B1/HSPA5/UPP1/XBP1 |
| GO:0051235 | maintenance of location | 0.001022 | CALR/FTL/HSP90B1/HSPA5/PDIA3/SUN3 |
| GO:0051651 | maintenance of location in cell | 0.001532 | FTL/HSPA5/PDIA3/SUN3 |
| GO:0031669 | cellular response to nutrient levels | 0.001718 | ATF3/HSP90B1/HSPA5/UPP1/XBP1 |
| GO:0042149 | cellular response to glucose starvation | 0.002197 | HSPA5/UPP1/XBP1 |
| GO:2001233 | regulation of apoptotic signaling pathway | 0.002393 | ARHGEF2/ATF3/HERPUD1/PDIA3/SERPINE1/XBP1 |
| GO:0031668 | cellular response to extracellular stimulus | 0.002673 | ATF3/HSP90B1/HSPA5/UPP1/XBP1 |
| GO:1903573 | negative regulation of response to endoplasmic reticulum stress | 0.002918 | HERPUD1/HSPA5/XBP1 |
| GO:0009267 | cellular response to starvation | 0.004569 | ATF3/HSPA5/UPP1/XBP1 |
| GO:0031667 | response to nutrient levels | 0.005673 | ATF3/HSP90B1/HSPA5/SPX/UPP1/XBP1 |
| GO:2001236 | regulation of extrinsic apoptotic signaling pathway | 0.005975 | ARHGEF2/ATF3/PDIA3/SERPINE1 |
| GO:0070059 | intrinsic apoptotic signaling pathway in response to endoplasmic reticulum stress | 0.005975 | HERPUD1/TRIB3/XBP1 |
| GO:1902041 | regulation of extrinsic apoptotic signaling pathway via death domain receptors | 0.005975 | ARHGEF2/ATF3/SERPINE1 |
| GO:1990440 | positive regulation of transcription from RNA polymerase II promoter in response to endoplasmic reticulum stress | 0.006449 | ATF3/HSPA5 |
| GO:0009991 | response to extracellular stimulus | 0.006449 | ATF3/HSP90B1/HSPA5/SPX/UPP1/XBP1 |
| GO:0061042 | vascular wound healing | 0.007172 | SERPINE1/XBP1 |
| GO:0035437 | maintenance of protein localization in endoplasmic reticulum | 0.007997 | HSPA5/PDIA3 |
| GO:0042594 | response to starvation | 0.007997 | ATF3/HSPA5/UPP1/XBP1 |
| GO:0030433 | ubiquitin-dependent ERAD pathway | 0.009369 | HERPUD1/HSP90B1/HSPA5 |
| GO:0032507 | ER-nucleus signaling pathway | 0.009433 | HSPA5/PDIA3/SUN3 |

Table S4: Summary the expression of several genes affected by FA deficiency in apoptotic pathway and cancer pathway. (The result from mRNA sequencing)

| Gene ID | Symbol | log2 Fold Change | P value |
| --- | --- | --- | --- |
| 472 | ATM | 0.263444 | 0.455789 |
| 581 | BAX | -0.155706 | 0.599417 |
| 1499 | CTNNB1 | 0.065345 | 0.814904 |
| 4193 | MDM2 | -0.115529 | 0.690841 |
| 1026 | CDKN1A | -0.625668 | 0.030843 |
| 7157 | TP53 | -0.599846 | 0.051260 |
| 7422 | VEGFA | -0.539927 | 0.062468 |


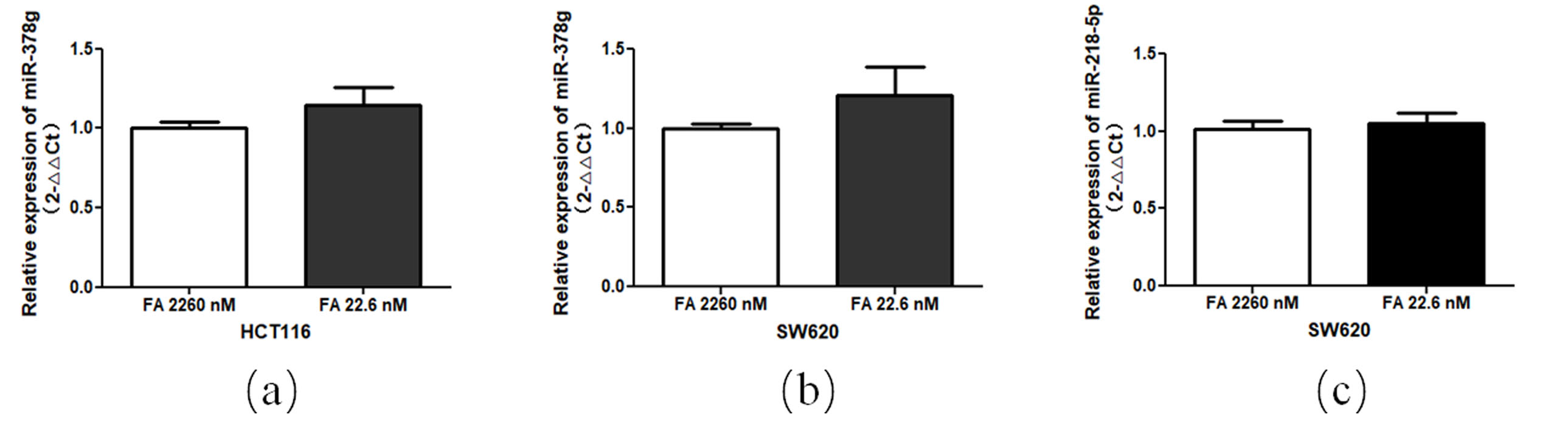


Figure S1. The supplemental RT-qPCR result of miRNAs in HCT116 and SW620 in FA deficiency conditions. (a) miR-378g in HCT116; miR-378g (b) and miR218-5p (c) in SW620. Bars represent standard errors.


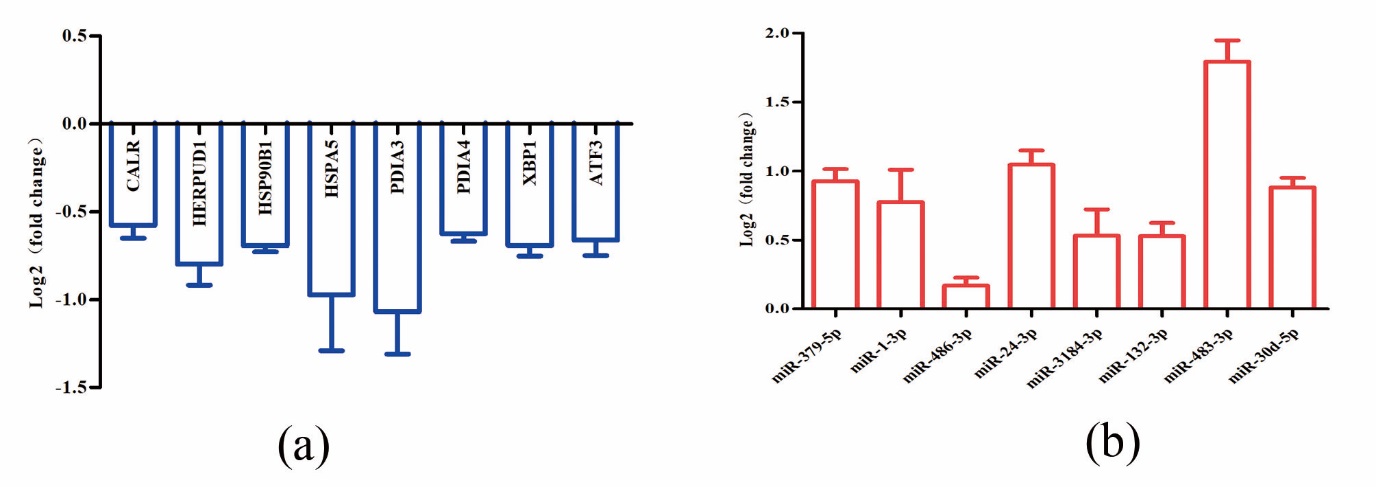


Figure S2. The RT-qPCR result of SW620 under the same FA deficiency conditions. (a) mRNAs. (b) miRNAs.The height of the columns in the chart represents the log-transformed average fold change (deficient FA / adequate FA). All columns have statistical significance (p<0.05) in this diagram. Bars represent standard errors.
